# MicroRNA-127 targeting of mitoNEET inhibits neurite outgrowth, induces cell apoptosis and contributes to physiological dysfunction after spinal cord transection

**DOI:** 10.1038/srep35205

**Published:** 2016-10-17

**Authors:** Qin-Qin He, Liu-Lin Xiong, Fei Liu, Xiang He, Guo-Ying Feng, Fei-Fei Shang, Qing-Jie Xia, You-Cui Wang, De-Lu Qiu, Chao-Zhi Luo, Jia Liu, Ting-Hua Wang

**Affiliations:** 1 Institute of Neurological Disease, Department of Anesthesiology and Translational Neuroscience Center, the state key laboratory of Biotherapy, West China Hospital, Sichuan University, Kunming 650500, China; 2 Institute of Neuroscience, Animal Zoology Department, Kunming medical University, Kunming 650500, China; 3 Department of Histology and Embryology, West China School of Preclinical and Forensic Medicine, Sichuan University, Chengdu 610041, China; 4 Department of Anesthesiology, Chengdu fifth people’s hospital, Chengdu 610031, China

## Abstract

Neuroregeneration and apoptosis are two important pathophysiologic changes after spinal cord injury (SCI), but their underlying mechanisms remain unclear. MicroRNAs (miRNAs) play a crucial role in the regulation of neuroregeneration and neuronal apoptosis, research areas that have been greatly expanded in recent years. Here, using miRNA arrays to profile miRNA transcriptomes, we demonstrated that miR-127-3p was significantly down-regulated after spinal cord transection (SCT). Then, bioinformatics analyses and experimental detection showed that miR-127-3p exhibited specific effects on the regulation of neurite outgrowth and the induction of neuronal apoptosis by regulating the expression of the mitochondrial membrane protein mitoNEET. Moreover, knockdown of MitoNEET leaded to neuronal loss and apoptosis in primary cultured spinal neurons. This study therefore revealed that miR-127-3p, which targets mitoNEET, plays a vital role in regulating neurite outgrowth and neuronal apoptosis after SCT. Thus, modificatioin of the mitoNEET expression, such as mitoNEET activition may provide a new strategy for the treatment of SCI in preclinical trials.

Spinal cord injury (SCI) remains a great challenge to clinicians and imposes a hefty financial burden on economies across the world. Tribulations have been encountered along the way owing to the complicated set of pathways that are initiated post-injury^1^. The neuropathology of SCI includes mitochondrial dysfunction and multiple cellular and biochemical reactions that lead to neuroinflammation, neurodegeneration and apoptosis^2^,^3^,^4^,^5^. MiRNAs, 19- to 23-nucleotide non-coding small RNA molecules, are implicated in a variety of biological processes, including neuron plasticity, apoptosis and mitochondrial functions^6^,^7^, in the central nervous system (CNS). MiRNA targets included signaling networks that were associated with pathological responses after SCI, including SCT, which could become attractive novel therapeutic targets for the treatment of SCI^8^,^9^. The mechanisms underlying the effects of SCI are multifaceted, extremely complicated and still not completely understood. More extensive research is needed to determine the exact role of miRNAs in the neuroprotective effects after SCI.

In this study, we first performed comprehensive miRNA microarray analyses on transected spinal cord (SCT) versus normal spinal cord in adult SD rats. Then, qRT-PCR assays were performed on specific highlighted miRNAs to confirm the miRNA array findings. MiR-127 was one of the most down-regulated miRNAs after SCT. Furthermore, we demonstrated that miR-127 agomir exacerbated motor functional deficits, inhibited neural plasticity, and increased cell apoptosis after SCT. Gain of miR-127 function using miR-127 mimic inhibited axon regeneration and increased neuronal apoptosis in spinal neurons. Furthermore, we found that the mitochondrial membrane protein mitoNEET was a direct target of miR-127 in neurons. MitoNEET, which serves as an iron-sulfur cluster transfer protein that binds to the mitochondrial outer membrane^10^, is essential for maximal energy production and exertion of mitochondrial activity^11^,^12^. Current evidence demonstrates that mitoNEET is proposed to mediate iron and reactive oxygen homeostasis in mitochondria, which is linked to mitochondrial injury and cell death in cancer therapy^13^. The literature shows that mitoNEET could ameliorate the mitochondrial dysfunction and inhibit cell death pathways following SCI^14^. Therefore, the administration of mitoNEET could be considered a candidate therapeutic strategy for the repair of SCI.

Moreover, we confirmed that knockdown of mitoNEET could induce the apoptosis of primary cultured spinal neurons and inhibited axonal growth, as indicated by the down-regulation of GAP-43. Importantly, miR-127 could negatively regulate neurite outgrowth and promoted cell apoptosis. The underlying mechanism involves the targeting of the mitochondrial membrane protein mitoNEET to the injured spinal cord.

## Results

### MicroRNA expression patterns after SCT

**Figure 1:**
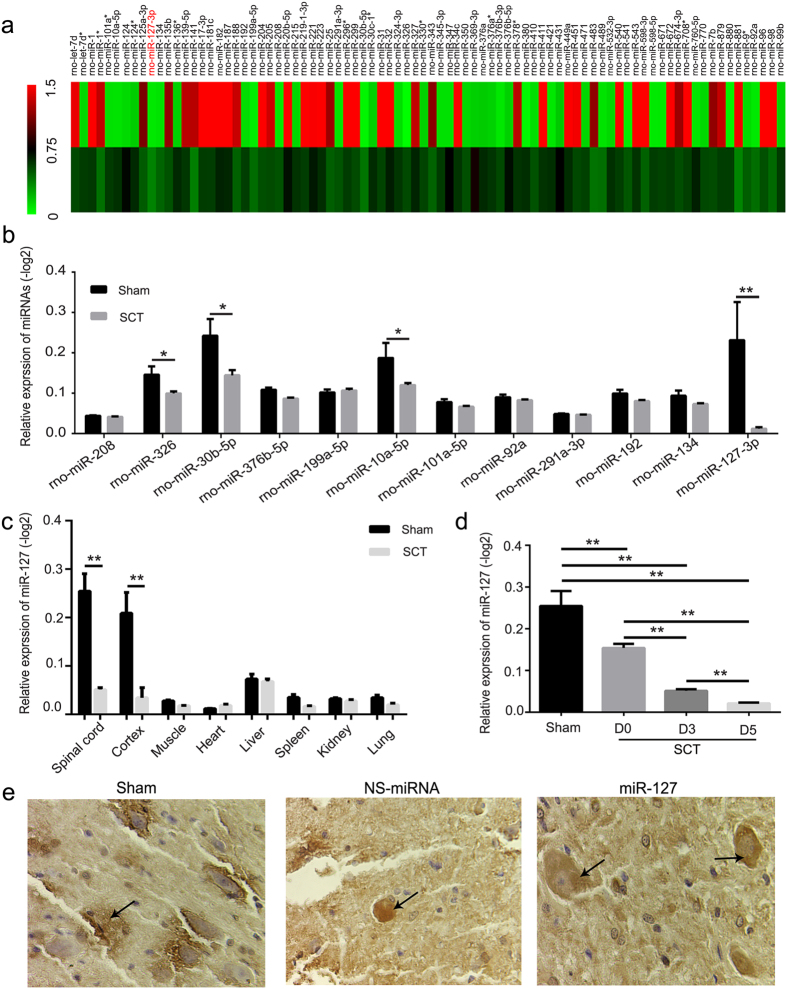
MicroRNA-127 was one of the spinal cord enriched miRNAs with a significant down-regulation after SCT. (**a**) 3 dpo, forty-two microRNAs were down-regulated 2-fold and forty-two microRNAs were up-regulated 2-fold after SCT compared to the sham group, which were normalized to −log 2 and plotted as a heat map. The color code in the heat maps is linear, with green as the lowest and red as the highest. (**b**) 3 dpo, miRNA array data were verified by qRT-PCR. The fold change values indicated the relative change in the expressional levels between samples and the internal control (U6), assuming that the value of the U6 expression level of each sample was equal to 1. And expression of miRNA in each group was presented as mean ± SE relative to −log2. n = 5/group. (**c**) qRT-PCR analysis of miR-127 expression in spinal cord, cortex, liver, muscle, spleen, kidney, heart and lung using total RNA isolated from the sham and SCT rats. Each bar is the mean ± SE relative to −log2. *P < 0.05, **P < 0.01. n = 5/group. (**d**) qRT-PCR analysis of miR-127 expression at D0, D3 and D5. **P < 0.01. n = 5/group. D0, D3 and D5 represent day 0, day 3 and day 5 post operation. (**e**) The localization of miR-127 in spinal cord tissue was detected by *in situ* hybridization 28 days after SCT. MiR-127 was localized in the cytoplasm of neurons following SCT (arrows). Scale bar = 50 μm.

To investigate the role of miRNAs in injured spinal cords, we first examined changes in miRNA expression after SCT using miRNA microarrays. We analyzed the miRNA expression patterns 3 days after transected SCT. The microarray results revealed that forty-two miRNAs were down 2-fold and forty-two miRNAs were up-regulated more than two times after SCT compared with the sham group (Fig. 1a). To further confirm the microarray data, twelve highly conserved miRNAs in vertebrates were selected, and qRT-PCR analysis was performed (Fig. 1b). Consistent with the miRNA array data, the qRT-PCR analysis indicated that miR-326, miR-30b-5p, miR-10a-5p and miR-127-3p were down-regulated more than four-fold after SCT (versus sham group, P < 0.02) (Fig. 1b). Additionally, miR-127 was one of the most significantly down-regulated miRNAs after SCT (versus sham group, p = 0.012) (Fig. 1b,c). Moreover, miR-127 was expressed at a low level in liver, muscle, spleen, kidney, heart and lung but at a relatively high level in the spinal cord and cortex in normal rats (Fig. 1c). In order to ensure the accuracy of the changing trend for miR-127, we dynamically validated the expression of miR-127 at D0, D3 and D5 by qRT-PCR. The results showed that miR-127 was gradually decreased with the time interval (P < 0.01). *In situ* hybridization indicated that miR-127 was clearly observed in neurons in spinal cord tissue (Fig. 1e). This result indicated that miR-127 was one of the CNS-enriched miRNAs and highly expressed in the spinal cord, while it was significantly down-regulated after SCT (Fig. 1). In addition, in order to demonstate the research value of miR-127, we also summarized and compared our miRNAs with other previously reported miRNAs in spinal cord injury model (see Sup Table).

### MiR-127 exacerbated the motor and sensory functional deficits induced by SCT

#### BBB locomotor rating.

**Figure 2:**
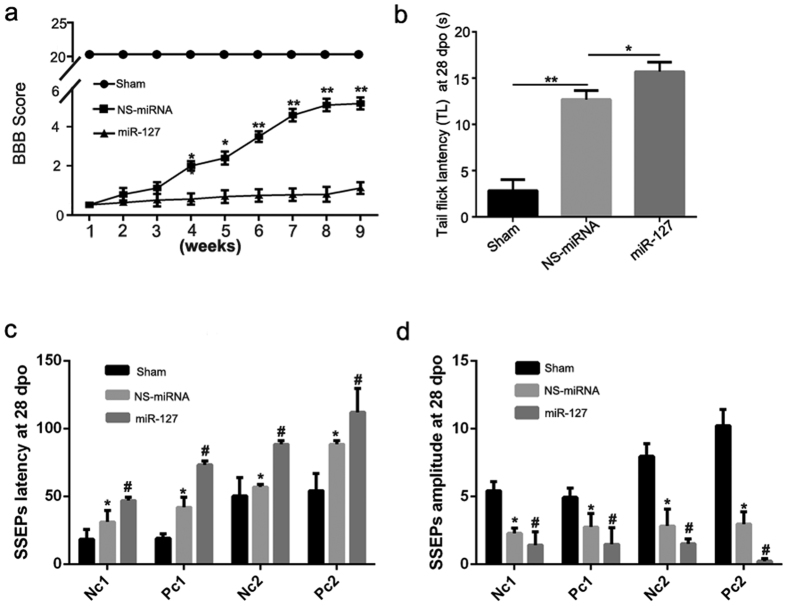
miR-127 exacerbated dysfunction after SCT. (**a**) miR-127 exacerbated the locomotor function deficit following SCT. Functional recovery in hindlimb was assessed from week 1 to week 9 after SCT with Basso, Beattie, and Bresnahan (BBB) Scores. Hindlimb dysfunction was exacerbated with treatment of miR-127 agomir when compared with NS-miRNA group. Three independent experiments were performed and the data are presented as the means ± SEM. *P < 0.05, **P < 0.01, compared with NS-miRNA group. n = 5 at least/group. (**b**) Tail-flick latency (TL) in each group was evaluated at 28 days after SCT. Data were presented as means ± SEM. *P < 0.05, **P < 0.01, compared with NS-miRNA group. n = 5/group. (**c,d**) Latency and amplitude of SSEPs signals in each group was evaluated at 28 days after SCT. Data were presented as means ± SEM. *P < 0.05 versus sham group, ^#^P < 0.05 versus NS-miRNA group. n = 5/group.

BBB scoring was performed to assess changes in gross locomotor performance following SCT (Fig. 2a). Prior to transection surgery, all the animals achieved a baseline score of 21, indicating normal locomotion and hindlimb function. Locomotor function was most severely impaired post-injury, with a mean BBB score of 0 on the 1^st^ day post-injury (representing total paralysis in both hindlimbs). Some spontaneous recovery was observed at the 4^th^ week post-injury, with mean BBB scores of 2 ± 0.3 in NS-miRNA group and 0.8 ± 0.02 in miR-127 group. Compared with the NS-miRNA group, locomotor function recovery was significantly attenuated in the miR-127 agomir group from the 4^th^ week post-injury (the 4^th^, 5^th^ week, p < 0.05; the 6^th^ week, 7^th^ week, 8^th^ week and 9^th^ week, p < 0.01).

### Tail-flick latency

At 28 days after being treated with miR-127 agomir, rats exhibited impaired thermal sensory function (Fig. 2b). A significant prolongation of tail-flick latency was detected 28 days after treatment with miR-127 agomir at the level of segments T9 and T11 (versus. NS-miRNA group, P = 0.037) (Fig. 2b).

### Somatosensory evoked potentials (SSEPs)

The normal SSEP wave form of the nervus peroneus communis in rats consists of four major identifiable components, i.e., Nc1, Pc1, Nc2, and Pc2 (data not shown). Compared with the sham group, there was a significant prolongation of the latencies of SSEP signals in the NS-miRNA group, thus Nc1, Pc1, Nc2 and Pc2 in the left hindlimb (P < 0.05) (Fig. 2c). In the NS-miRNA group, the amplitudes of Nc1, Pc1, Nc2 and Pc2 in the left hindlimb were also reduced (versus sham group, P < 0.05) (Fig. 2d). In the miR-127 group, there was a significant prolongation of the latencies of SSEP signals—Nc1, Pc1, Nc2 and Pc2 (versus the NS-miRNA group, P < 0.05) (Fig. 2c). The amplitudes of Nc1, Pc1, Nc2 and Pc2 in the miR-127 group were reduced as well (versus the NS-miRNA group, P < 0.05) (Fig. 2d).

### MiR-127 induced neuron loss, axonal degeneration and cell apoptosis after SCT

**Figure 3:**
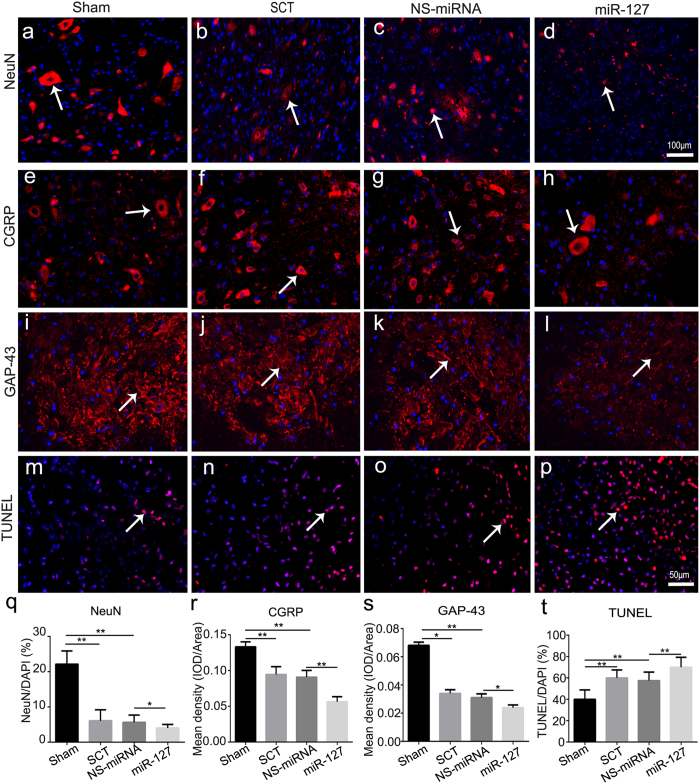
MiR-127 increased neuronal loss, promoted cell apoptosis and inhibited axonal regeneration after SCT. Slices of rostral spinal cord derived from 28 dpo were subjected to immunohistochemistry for NeuN (**a**–**d**, red, white arrow), CGRP (**e**–**h**, red, white arrow), GAP-43 (**i**–**l**, red, white arrow), and counterstained with DAPI (blue) in sham group (**a,e,i**, n = 5), SCT group (**b,f,j**, n = 5), NS-miRNA (**c,g,k**, n = 5) and miR-127 group (**d,h,l**, n = 5). Three days post operation, TUNEL staining was used to analyze neuronal apoptosis (**m**–**p**, red, white arrow) in rostral of spinal cord in sham, SCT, NS-miRNA and miR-127 group. Sections were stained with DAPI (blue) to show all nuclei, and TUNEL (red, white arrow) to show apoptotic cells, in merged photomicrographs rose-red were defined as TUNEL positive. (**q**) The percentage of the NeuN^+^/DAPI was measured. (**r,s**) Mean density of CGRP (**r**) and GAP-43 (**s**), which presented as IOD/Area in each group were measured. (**t**) Quantitative histogram showed the percentage of TUNEL/DAPI in sham, SCT, NS-miRNA and miR-127 group. *P < 0.05, **P < 0.01. Scale bar: (**a–d**), 100 μm; (**e–p**), 50 μm.

At 28 days after SCT, there was a significant loss of neurons (Fig. 3b) and decreases in CGRP (Fig. 3f) and GAP-43 (Fig. 3j) in the SCT group compared to the sham group (Fig. 3a,e,i). In miR-127 group, the NeuN-positive cell counts (Fig. 3d) and the mean densities of CGRP (Fig. 3h) and GAP-43 (Fig. 3l) were significantly decreased compared with the NS-miRNA group (Fig. 3c,g,k, P < 0.05), whereas no difference in gliosis was detected in miR-127 group (versus NS-miRNA group, P > 0.05) (Fig. S2c,d). Consistent with the observed neuron loss, TUNEL-positive nuclei in the miR-127 group (Fig. 3p) were markedly increased (versus. the NS-miRNA group, Fig. 3o, P = 0.014) (Fig. 3t). Taken together, these results indicate that miR-127 inhibited axonal generation and enhanced cell apoptosis after SCT.

### MiR-127 induced neuronal loss, neurodegeneration, and neuronal apoptosis in primary cultured spinal neurons

**Figure 4:**
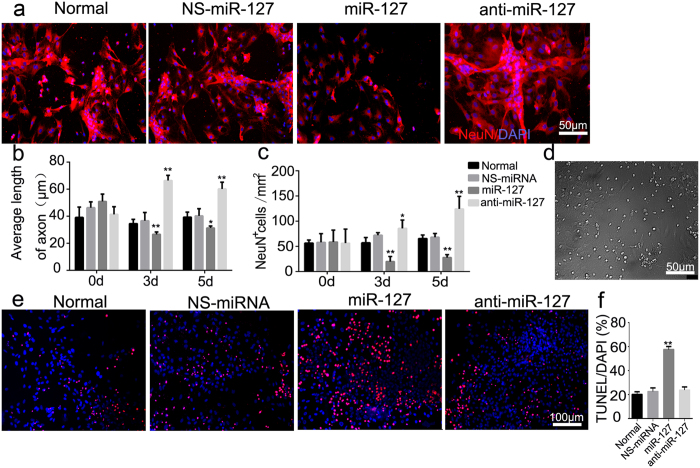
MiR-127 increased neural loss, inhibited axonal regeneration and increased apoptosis of primary cultured spinal neurons. (**a**) NeuN immunoreactive staining in normal, NS-miRNA (80 nM), miRNA-127 mimic (80 nM) and anti-miR-127 (100 nM) group. Red signal represented NeuN-positive neurons and blue signal represented nucleus of all cell types. All imaging pictures were taken at 3 days after transfection. (**b**) Average length of axon in Normal, NS-miRNA, miR-127 mimic and anti-miR-127 group was measured by using Leica AF6000 cell station. Graphs represent the mean ± SEM of quintuplicate culture dishes per group at each time point from three separate experiments. *P < 0.05, **P < 0.01, compared at different time point in the same group. ^#^P < 0.05, ^##^P < 0.01 compared with the NS-miRNA group at the same time point. (**c**) Average number of NeuN positive cells per mm^2^ after being transfected with miR-127 mimic or anti-miR-127 was measured. Graphs represent the mean ± SEM of quintuplicate culture dishes per group at each time point from three separate experiments. *P < 0.05, **P < 0.01, compared at different time point in the same group. ^#^P < 0.05, ^##^P < 0.01 compared with the NS-miRNA group at the same time point. (**d**) Representative bright field picture of the primary cultured spinal neurons at 5 days after being transfected with NS-miRNA. (**e**) TUNEL staining was used to analyze neuronal apoptosis (red fluorescence, white arrow) in normal, NS-miRNA (80 nM), miRNA-127 mimic (80 nM) and anti-miR-127 (100 nM) group at 3 days after transfection. n = 5/group. (**f**) Percentage of TUNEL/DAPI was shown in normal group, NS-miRNA, miR-127 mimic, and anti-miR-127 group. *P < 0.05, **P < 0.01, compared with the NS-miRNA group. Scale bar: (**a,d**) 50 μm; (**e**) 100 μm.

NeuN-positive cells were observed in the normal, NS-miRNA, miR-127 and anti-miR-127 groups (Fig. 4a). In the presence of a high level of miR-127, the average axonal length was decreased both 3 and 5 days after miR-127 transfection, compared with the NS-miRNA group (P < 0.05), whereas the neurons transfected with anti-miR-127 presented a longer axonal length at 3 and 5 days after transfection (versus the NS-miRNA group, P < 0.01) (Fig. 4b,d). At 3 and 5 days after transfection, the number of NeuN^+^ cells were significantly decreased in the miR-127 group (versus the NS-miRNA group, P < 0.05) (Fig. 4c), whereas in the anti-miR-127 group, NeuN^+^ cells were increased (versus the NS-miRNA group, P < 0.05) (Fig. 4c). Moreover, miR-127 had no effect on the cell counts of astrocytes (Fig. S2a,b). TUNEL-positive cells were observed in the normal, NS-miRNA, miR-127 and anti-miR-127 groups (Fig. 4e). Apoptosis of primary cultured spinal neurons was increased more than two-fold in the miR-127 group compared to the NS-miRNA group (P < 0.01) (Fig. 4f).

There was no significant difference of the percentage of TUNEL/DAPI between the anti-miR-127 group and NS-miRNA group (P > 0.05) (Fig. 4f).

### MiR-127 modulated mitoNEET rather than KCC1 mRNA and protein expression

**Figure 5:**
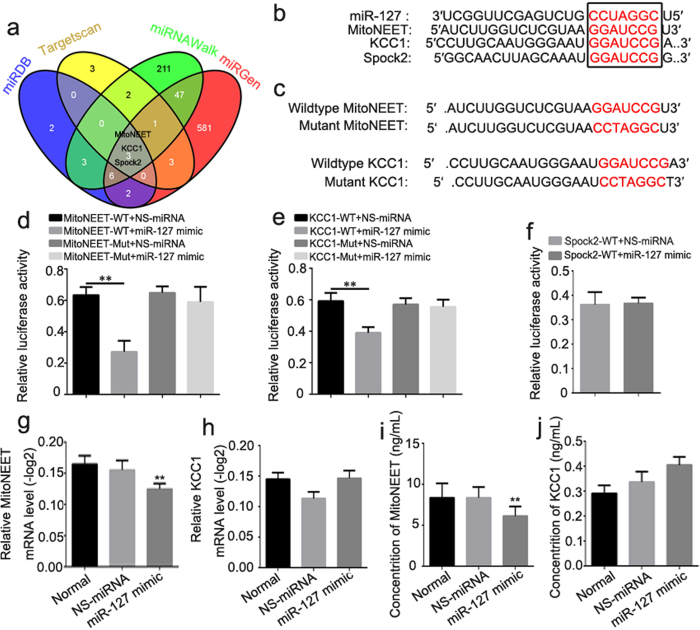
MitoNEET is one of the direct targets of miR-127. (**a**) Venn diagram of target genes of miR-127 which derived from conventional online programs of miRDB, TargetScan, miRNAWalk and miRGen. MitoNEET, KCC1 and Spock2 are the common targets of miR-127 in the four conventional online programs which were selected for further investigation. (**b**) The alignment of the seed regions of miR-127 with MitoNEET, KCC1 and Spock2 3′-UTR. (**c**) Alignment of the seed regions of miR-127 with MitoNEET and KCC1 3′ UTR, and the mutation of MitoNEET and KCC1 3′ UTR sequence in the complementary site. (**d,e,f**) 293Tα cells were co-transfected with 80 nM of miR-127 mimic or NS-miRNA and 100 ng/ml of 3′-UTR reporter plasmid of MitoNEET, KCC1 and Spock2 or the relative mutant form. Luciferase activity was detected at 48 h after transfection. Relative luciferase activity was calculated with (Rluc miRNA/hLuc miRNA)/(Rluc NS-miRNA/hLuc NS-miRNA). (**g,h**) Total RNA of primary spinal neurons was extracted and performed for qRT-PCR of MitoNEET (**g**) and KCC1 (**h**) 72 h after being transfected with miR-127 mimic or NS-miRNA. (**i,j**) The protein expression of MitoNEET (**i**) and KCC1 (**j**) in primary spinal neurons was detected by ELISA 72 h after being transfected with miR-127 mimic or NS-miRNA. *P < 0.05, **P < 0.01 compared with NS-miRNA.

**Figure 6:**
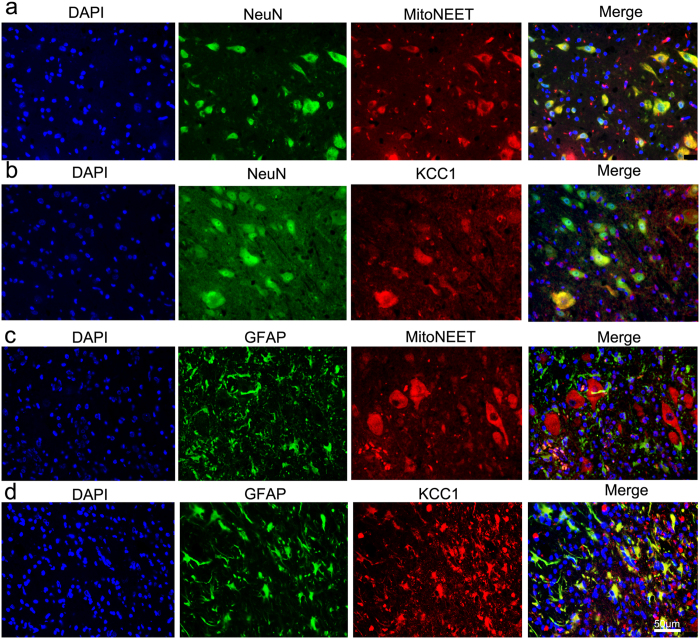
The localization of MitoNEET and KCC1 in spinal cord. (**a,b**) Double-label fluorescence detection of NeuN/MitoNEET (**a**) and NeuN/KCC1 (**b**) were carried out in spinal cord. Sections were stained with DAPI (**a**,**b**, blue, the first panel) to show all nuclei, NenN (**a**,**b**, green, the second panel), MitoNEET (**a**, red, the third panel), KCC1 (**b**, red, the third panel), and the merge image (**a,b**, the last panel). The merge image shows the region of co-localization appearing yellow. (**c,d**) Double-label fluorescence detection of GFAP/MitoNEET (**c**) and GFAP/KCC1 (**d**) were carried out in spinal cord. Sections were stained with DAPI (**c**,**d**, blue, the first panel) to show all nuclei, GFAP (**c**,**d**, green, the second panel), MitoNEET (**c**, red, the third panel), KCC1 (**d**, red, the third panel), and the merge image (**c**,**d**, the last panel). The merge image shows the region of co-localization appearing yellow. Scale bar: (**a–d**) 50 μm.

To investigate how miR-127 participates in the regulation of neural regeneration, we searched for miR-127 target genes in primary spinal neurons. Three genes identified by all four programs (miRDB, TargetScan, miRNAWalk and miRGen) (Fig. 5a) were selected for further investigation: MitoNEET, KCC1 and Spock2, which are related to mitochondrial dysfunction, cell edema and synapse biogenesis, respectively. The 3′-UTRs of these three genes were cloned into the pmi-R-RB-REPORT plasmid (the predicted binding sites for miR-127 are shown in Fig. 5b). The mutated construct was generated using site-directed mutagenesis of a reporter containing the 3′-UTRs of the MitoNEET and KCC1 genes (Fig. 5c). In cells transfected with plasmids containing the 3′-UTRs of MitoNEET (Fig. 5d) and KCC1 (Fig. 5e), the relative luciferase activity was significantly decreased after treatment with miR-127, whereas the inhibitory effect of miR-127 was abolished in the plasmid containing the mutant 3′-UTRs of MitoNEET (Fig. 5d) and KCC1 (Fig. 5e). The relative luciferase activity was not obviously altered in cells transfected with a plasmid containing the 3′-UTR of Spock2 (Fig. 5f). qRT-PCR and ELISA analyses revealed that the mRNA and protein expression of MitoNEET, but not KCC1, was impaired by treatment with miR-127 in primary spinal neurons (Fig. 5g,h,i,j). Double immunofluorescence demonstrated that MitoNEET was localized to neurons rather than astrocytes (Fig. 6a,c), whereas KCC1 was ubiquitously expressed in neurons and astrocytes (Fig. 6b,d). Together, our results demonstrate that MitoNEET is a major direct target of miR-127 in spinal neurons.

### Knockdown of MitoNEET induced neuronal loss and neuronal apoptosis in primary cultured spinal neurons

**Figure 7:**
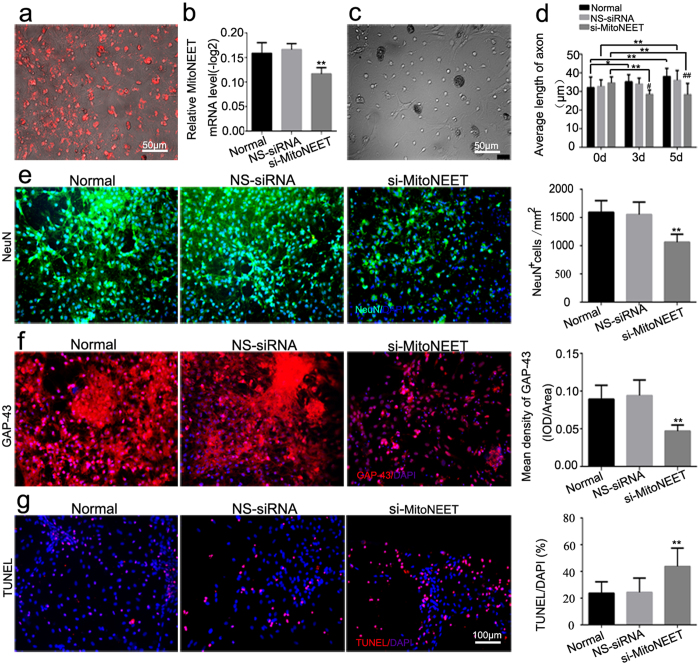
si-MitoNEET increased neural losses, inhibited axonal regeneration, and promoted neural apoptosis. (**a**) Transfected Cy3-5′-si-MitoNEET with red fluorescence was observed in primary spinal neurons at 48 h after transfection. (**b**) Total RNA of primary cultured spinal neurons was extracted and qRT-PCR of MitoNEET was performed in normal, NS-siRNA (100 nM) and si-MitoNEET (100 nM) group 72 h after transfection. (**c**) Representative bright field picture of the primary cultured spinal neurons 5 days after transfection with NS-siRNA. (**d**) Average length of axon spinal neurons was measured by using Leica AF6000 cell station. Data are presented as the means ± SEM. *P < 0.05, **P < 0.01, compared at different time point in the same group. ^#^P < 0.05, ^##^P < 0.01 compared with the NS-siRNA group at the same time point. (**e**) Immunostaining of NeuN in normal, NS-siRNA (100 nM) and si-MitoNEET (100 nM) group was performed 3 days after the transfection. Green signal represented NeuN-positive neuron and blue signal represented nucleus of all cell types (left). And average number of NeuN positive cells per mm^2^ was measured (right). (**f**) Immunofluorescence staining of GAP-43 (red, left) in normal, NS-siRNA and si-MitoNEET group was performed. DAPI (blue) was employed to show all nuclei. Mean density of GAP-43, which presented as IOD/Area in each group was measured (right). (**g**) TUNEL staining (white arrow) was performed in normal, NS-siRNA (100 nM) and si-MitoNEET (100 nM) group at 3 days after transfection. DAPI (blue) was used to show all nuclei. The percent of TUNEL/DAPI was evaluated and indicated by quantitative histogram (right). *P < 0.05, **P < 0.01, compared with NS-siRNA. Scale bar: (**a,c**) 50 μm; (**e**–**g**) 100 μm.

Red fluorescently labeled cy3-5′-si-MitoNEET indicated that a MitoNEET knockdown could successfully be used to transfect primary spinal neurons (Fig. 7a). Three small interfering RNAs of MitoNEET and KCC1 were designed and synthesized. The mRNA expression of MitoNEET and KCC1 was obviously decreased in PC12 cells transfected with si-r-KCC1-001 and si-r-MitoNEET-001 (versus NS-siRNA, P < 0.05) (Fig. S1). Moreover, the mRNA levels of MitoNEET and KCC1 in primary cultured spinal neurons were significantly decreased after interference of MitoNEET (Fig. 7b) and KCC1 (Fig. S3a). Significant reductions in both neuron numbers and axonal length were observed after transfection with si-MitoNEET compared with NS-siRNA (P < 0.05) (Fig. 7c,d,e). Consistent with the observed neuroregeneration inhibition in the miR-127 group, the signs of neuroregeneration by immunohistochemical analysis of GAP-43 were decreased more than two-fold by interference of MitoNEET in primary cultured spinal neurons (versus NS-siRNA, P < 0.05) (Fig. 7f). The percentage of TUNEL/DAPI was increased more than two-fold after MitoNEET interference compared with the NS-siRNA group (P < 0.05, Fig. 7g). No obvious changes were observed in neuron counts, axonal length, the mean density of GAP-43 or the percentage of TUNEL/DAPI between the si-KCC1 group and the NS-siRNA group (P > 0.05, Fig. S3b,c,d,e,f).

## Discussion

In this study, we found that miR-127 exacerbated the motor and sensory functional deficits in SCT rats. Furthermore, we showed that miR-127 inhibited neurite outgrowth and promoted cell apoptosis. Then, we confirmed that the mitochondrial membrane protein mitoNEET was a direct target of miR-127, which affected key processes in the repair and regeneration of SCT. Briefly, in our study, we got three conclusions: 1. miR-127 activation exacerbated behavioral dysfunction, impeded the axonal growth and promoted neural apoptosis.2. Knockdown of mitoNEET inhibited axonal outgrowth and promoted neural apoptosis. 3. miR-127 can target mitoNEET.

MiR-127 is one of the CNS-enriched miRNAs and highly expressed in the spinal cord. MiR-127 has been reported to be downregulated in several cancers, including hepatocellular carcinoma, breast cancer and oral cancers, which suppressed cell growth or enhanced apoptosis^15^,^16^,^17^. In the central nervous system, miR-127 was observed to be a neuron-enriched miRNA that played a vital role in neuronal differentiation^18^,^19^. Nai-Kui Liu *et al.* reported that miR-127 was up-regulated as early as the 4^th^ hour post-SCI, followed by down-regulation on the 1^st^ day and the 7^th^ day post-SCI^20^. Some researchers have proposed that MiR-127 may participate in secondary pathogenesis after spinal cord injury^8^; however, this is not well understood. In our study, we found that miR-127 levels significantly decreased 3 days after SCT. Furthermore, we found that miR-127 exhibited low expression in the liver, muscle, spleen, kidney, heart and lung of rats but high expression in spinal cord and cortex, which indicated that miR-127 may be a CNS-enriched miRNA.

MiR-127 exacerbated the motor and sensory functional deficits and inhibited the nerve regeneration after SCT. BBB scale is a valid and predictive measure of locomotor recovery^21^. Constant with the previous study, we observed a spontaneous locomotor functional recovery in SCT rats^22^,^23^. However, BBB scale indicated an aggravated the motor dysfunction in SCT rats after being injected of miR-127 agomir. SSEPSs are responsive to sensory recovery and correlated with ambulatory capacity, which is predictive validity to measure the function rehabilitation after SCI^24^,^25^,^26^. In our study, SSEPs of rats presented a prolongation of the latency and a reduction of the amplitude after treatment with miR-127. Videlicet, the rehabilitation after SCT was inhibited after being treated with miR-127. Moreover, Tail-flick latency (TL) of thermal pain is a well characterized and simple model considered as a suitable parameter for predicting the algesia in humans and rats^27^,^28^. Our data demonstrated that there was hypalgesic in the rats with SCT, and this feeling dysfunction was further exacerbated after treatment with miR-127. Taken together, our study demonstrated that miR-127 exacerbated both motor and sensory functional deficit after SCT. Nerve reinnervation and functional recovery after SCI may be achieved by long-distance axonal regeneration and consequent synapse formation on the appropriate (pre-injury) target cells, short-distance axonal regeneration followed by synapse formation on neuronal elements to create relays to neuronal targets located distal to the lesion site, and sprouting of surviving axons to form synapses on suitable targets beyond the injury site^29^,^30^. In the central nervous system, GAP-43 plays a role in neurite outgrowth. GAP-43 expression is upregulated in developing and actively growing or regenerating neurons and during nerve regeneration. CGRP peptide plays a vital role in regenerative sensory axon sprouts. In regenerative sensory axon sprouts, CGRP, a neuropeptide confined to fine primary afferent terminals in laminae I and II in the dorsal horn of the spinal cord and implicated in pain transmission, was selected. Sham rats displayed CGRP immunoreaction products in outer laminae I and II, Lissauer’s tract, dorsal roots, and motor neurons in the ventral horn. In our study, we found low expression of GAP-43 and CGRP in transected spinal cords injected with miR-127 agomir. Moreover, there was a decrease in NeuN-positive cells and an increase in TUNEL-positive cells after SCT treatment with miR-127. Thus, the over-expression of miR-127 in transected spinal cords aggravated neuronal loss and inhibited synapse formation and axon regeneration at the spinal cord lesion site. Inhibition of axon outgrowth, reduction of primary spinal neurons and an increase in neuronal apoptosis were also found in primary cultured spinal neurons after exposure to miR-127 mimic. Moreover, axon outgrowth and neural loss were reversed by anti-miR-127. Taken together, our data indicate that miR-127 suppressed neural plasticity by the promotion of neural loss, inhibition of neurite outgrowth, reduction of synapse formation and enhancement of neuronal apoptosis.

MitoNEET served as one of the direct targets of miR-127 to regulate axonal outgrowth and population of spinal neurons. Luciferase and mutant assays indicated that both mitoNEET and KCC1 were targets of miR-127. The qRT-PCR analysis revealed that miR-127 had no effect on the expression of KCC1 mRNA, whereas MitoNEET mRNA expression was significantly impaired after primary spinal neurons were treated with miR-127 mimic. The changes in MitoNEET mRNA levels caused by the modulation of miR-127 were also reflected in MitoNEET protein expression (via immunoblotting and immunocytochemistry) in a target-specific manner. Our results demonstrated that the mitochondrial membrane protein mitoNEET was a direct target of miR-127 in spinal neurons. In spinal cord, double immunofluorescence demonstrated that MitoNEET was localized to neurons rather than astrocytes, whereas KCC1 was ubiquitously expressed in neurons and astrocytes.

To further extensively determine the exact roles of MitoNEET and KCC1 in spinal neurons, we constructed small interfering RNAs of MitoNEET and KCC1 and transfected them into primary spinal neurons. Although the magnitude of the siRNA knockdown is not so impressive, the effects of this siRNA were obvious in the primary cultured spinal neurons. In our study, neural numbers, axonal length and GAP-43 expression decreased significantly after transfection with si-MitoNEET, compared with NS-siRNA, and the percentage of TUNEL/DAPI was increased more than two-fold after MitoNEET interference. Similarly, previous studies have screened the most effective fragment of shRNA for α-synuclein, ERP29 and Netrin-1 in PC12 cell line, the interference efficiency is similar to this experiment, while the inhibitory effect was also confirmed obviously^31^,^32^,^33^. However, KCC1 interference had no effect on neurite outgrowth or cell apoptosis. It has been well established that mitochondrial dysfunction following SCI may be critical for the development of secondary pathophysiology and neuronal cell death^34^,^35^. In 2005, Colca *et al.* discovered that pioglitazone was able to bind to an unknown < 17-kDa mitochondrial protein, which they termed “mitoNEET”^36^. Some researchers have suggested that MitoNEET is a PPAR agonist that can increase mitochondrial bioenergetics following SCI^14^. Additionally, mitoNEET could also inhibit cell death pathways, leading to diminished cell death^37^.

MiR-127 can target the mitochondrial membrane protein mitoNEET to inhibit axon growth and enhance cell apoptosis. Therefore, we proposed that miR-127 or MitoNEET might be a potential novel drug target and chemotherapeutic for the treatment of spinal cord injury in clinical trials.

## Conclusion

MiR-127 activation in SCT exacerbated motor and sensory functional deficits, impeded axonal outgrowth, and promoted neuron apoptosis. MitoNEET was the direct target of miR-127 in inhibiting axon growth and promoting neuron apoptosis. Therefore, modulation of mitoNEET, such as activating mitoNEET action following SCT may be served as a treatment-target in the therapy of SCI and represent a novel repair strategy to reduce tissue damage and increase function recovery after SCT.

## Materials and Methods

### Experimental design

Experimental design and different parameters measured was shown in Table 1.

### Experiment 1

miRNA microarray analysis and microarray data confirmation. The miRNA expression patterns at the 3^rd^ day following rat SCI were analyzed with miRNA microarray. Rats were randomly divided into two groups: sham and SCT group, n = 3 per group. Then, qRT-PCR was performed to confirm the microarray data, in which, sham and SCT rats were used, n = 5 per group.

### Experiment 2

miR-127 experiments. *In vivo*, the SCI rats were randomly divided into four groups, including sham, SCT, SCT + nonspecific (NS)-miRNA as control group and SCT + miR-127 agomir group. In nonspecific (NS)-miRNA group and agomir-127 group, rats were subjected to SCT and treated with nonspecific (NS)-miRNA and agomir-127, respectively. At the scheduled time points, rats in both groups were euthanized with an overdose of 3.6% chloral hydrate (100 mg/kg). Subsequently, the spinal cord was exposed, and a 10 mm long segment of the spinal cord centered at the injury epicenter was harvested. The time of euthanasia was determined according to the different parameters measured: motor function was scored (Basso, Beattie, and Bresnahan [BBB]) for 9 weeks after SCT, n was at least 5 per group. Terminal deoxynucleotidyl transferase-mediated dUTP Nick End Labeling (TUNEL) was performed at the 3^rd^ day after SCT, n = 5 per group, immunohistochemistry staining at the 4^th^ week after SCT, n = 5 per group.

To explore the role of miR-127 *in vitro*, we administrated miR-127 mimic and miR-127 inhibitor in primary cultured spinal neurons to study the growth characteristics at 3 and 5 days after the transplantation, which including the neuron counts, axonal length and the TUNEL numbers after the transfection, n = 5 per group per condition.Furthermore, to explore the targets of miR-127, bioinformatics prediction and verification experiments known as luciferase and mutant test were carried out. Finally, to determine the role of targets of miR-127, knockdown of the target in primary cultured spinal neurons were employed to study the growth characteristics of the neurons at the 3^rd^ and 5^th^ day after transplantation, n = 5 per group per condition.

### Animal protocol

Adult female Sprague–Dawley (SD) rats (two months old), weighing 180–220 g, were provided by the Center of Experimental Animals of Sichuan University. All experiments including animal care, breeding, and testing procedures conformed to the suggestions for the care and use of laboratory animals promulgated by *the Ministry of Science and Technology of the People’s Republic of China,* and which were approved by the Animal Care and Use Committee of Sichuan University. Animals were housed in individual cages in a temperature- (21–25 °C) and humidity (45–50%)-controlled room with a 12-h light/dark cycle and ad libitum access to pellet chow and water.

### SCT model

Rats were anesthetized with 3.6% chloral hydrate (50 mg/kg, i.p.). Complete spinal cord transection was performed as described previously^38^,^39^,^40^. Briefly, laminectomy was performed at thoracic vertebra level 9–11 (T9-11) to expose the T10 spinal segment. A complete transverse cut of the spinal cord was performed at the T10 level, resulting in a gap of 1~2 mm with no tissue removed. The complete transection of the spinal cord was verified by histological study. Sham-injured animals underwent laminectomy only with no further spinal cord damage (Sham-control). After surgery, the dura was sutured and the muscle and skin were closed in layers. All rats received an intramuscular injection of penicillin (160,000 U/ml/d, Harbin Pharmaceutical Group) daily until 3 days after the operation. Gentle manual compression of the bladder was performed twice per day after SCT.

### MicroRNA array analysis

At 3 days after surgery, three samples per condition were pooled into one SCT pool and one sham pool for microarray analysis^41^,^42^,^43^,^44^. And each pool was on a different array chip. Briefly, a 10-mm-long spinal cord rostral to the transected site was harvested and fresh-frozen in liquid nitrogen. Total RNA was isolated with the miRNeasy mini kit (Qiagen, West Sussex, UK). All RNA species (including miRNAs) were efficiently recovered. The RNA quality and quantity were measured with a NanoDrop^®^ spectrophotometer (ND-1000, Nanodrop Technologies), and the RNA integrity was determined by gel electrophoresis. After RNA isolation from the samples, the miRCURY™ Hy3™/Hy5™ Power labeling kit (Exiqon, Vedbaek, Denmark) was used for miRNA labeling. One microgram of each sample was 3′-end-labeled with the Hy3™ fluorescent label using T4 RNA ligase by mixing 3.0 μL of RNA with 0.5 μl of CIP buffer and 0.5 μl of CIP. The mixture was incubated for 30 min at 37 °C and was terminated by incubation for 5 min at 95 °C. Then, 3.0 μL of labeling buffer, 1.5 μL of fluorescent label (Hy3™), 2.0 μL of dimethyl sulfoxide (DMSO), and 2.0 μL of labeling enzyme were added to the mixture. The labeling reaction was incubated for 1 h at 16 °C and terminated by incubation for 15 min at 65 °C. After the labeling procedure was stopped, the Hy3™-labeled samples were hybridized onto a miRCURY™ LNA Array (v.16.0) (Exiqon). The entire 25 μL mixture of Hy3™-labeled samples with 25 μL of hybridization buffer was first denatured for 2 min at 95 °C, incubated on ice for 2 min, and then hybridized to the microarray for 16 h to 20 h at 56 °C in a 12-Bay Hybridization System (Nimblegen Systems, Inc., Madison, WI). Following hybridization, the slides were retrieved, washed several times with wash buffer from the kit (Exiqon), and finally dried by centrifugation for 5 min at 400 rpm. Then, the slides were scanned with an Axon GenePix 4000B microarray scanner (Axon Instruments, Foster City, CA). The scanned images were then imported into GenePix Pro 6.0 software (Axon) for grid alignment and data extraction. Replicated miRNAs were averaged, and miRNAs with intensities ≥50 in all samples were chosen to calculate the normalization factor.

### MiRNA array verification

The microarray data were verified by qRT-PCR. Total RNA from the spinal cord was extracted with Trizol (Invitrogen, CA). RNA samples were first added to poly(A) Tailing with the miDETECTATrack^TM^ miRNA qRT-PCR Start Kit (Ribobio, Guangzhou, China), 1 μg of total RNA and 1 μl of poly(A) polymerase at 37 °C for 1 h. Then, cDNA was synthesized from 10 μL of poly (A) tailing product with 8 μL of Reverse Transcriptase (Promega, USA) and 2 μL of miDETECT A Track^TM^ Uni-RT Primer (Ribobio, Guangzhou, China), which reacted at 42 °C for 1 h and then was incubated at 72 °C for 10 min. qRT-PCR was performed in a 20 μl reaction volume with the following reagents: SYBR Green Mix (10 μL of SybrGreen I mix, 0.5 μL of miDETECTATrack^TM^ miRNA Forward Primer forward and reverse primers (10 μM), and 2 μL of cDNA template) on an iQTM5 multicolor real-time PCR detection system (Bio-Rad, Hercules, CA, USA) with the following protocol: 95 °C for 3 min and 40 cycles of 95 °C for 10 s, 60 °C for 20 s, and 70 °C for 1 s. The relative miRNA expression was determined by calculating the mean difference between cycles thresholds of the miRNA from the U6 normalized control. The relative quantity of each miRNA was described using 2^−ΔC^_T_, where ΔC_T_ = (C_T_ miRNA-C_T_ U6). Sample means that were greater than ±2 standard deviations from the mean ΔCT after exclusion were considered outliers and removed from the analysis. Furthermore, qRT-PCR of miR-127 in spinal cord, cortex, liver, muscle, spleen, kidney, heart and lung were especially performed.

### MiR-127 micro injection

Rats were anesthetized with 3.6% chloral hydrate (50 mg/kg, i.p.), and then laminectomy was performed at the T10 level as previously described. MiR-127 was delivered into the gray matter of the spinal cord by the insertion of a pored glass pipette attached to a micro-injector carrying a capillary glass microelectrode (Thermo Scientific, Rockford, IL, USA). The target areas for injection included four total sites with 2 mm depth at the following coordinates: two sites 2.5 mm rostral and two sites 2.5 mm caudal to the injury site. Each injection site was injected with 1 μl of 0.25 nmol (nM) micrONTMrno-miR-127-3p agomir (RiboBio, China, Lot 8001) or nonspecific (NS)-miRNA (RiboBio, China, Lot 0312). Infusion was performed at a rate of 100 nl/min. After injection, the glass pipette was left in place for an additional 2 min before being slowly retracted.

### Basso, Beattie, and Bresnehan (BBB) score

Hindlimb locomotor functions of rats treated with/without SCT were evaluated with the BBB locomotor rating in an open enclosure (99 cm diameter, 23 cm depth) with scores graded from 0 points (absence of any hindlimb movement) to 21 points (normal mobility)^21^. In this study, locomotor behavior testing was performed weekly thereafter for 9 weeks. Baseline motor function was assessed on the day following surgery. Because rodents often remain motionless when introduced to a new apparatus, the subjects were acclimated to the observation fields for 5 min per day for 3 days prior to surgery. Each subject was placed in the open field, observed for 4 min, and scored for locomotor behavior. Care was taken to ensure that the investigators’ scoring behavior had high intra- and inter-observer reliability and that the investigators were blind to the subject’s experimental treatment.

### Electrophysiological evaluation of ascending somatosensory evoked potentials (SSEPs)

Prior to each SSEP recording, rats were anesthetized for approximately 30 min as previously described. An isolated constant current stimulator (DS3, Digitimer Ltd., Hertfordshire, England) with subcutaneous needle electrodes (Safelead F-E3-48, Grass-Telefactor, West Warwick, RI) were used for electrical stimulation of hindlimb. A needle electrode was inserted into the left hindlimb to stimulate the common peroneal nerve (CPN) without directly contacting the nerve bundle.Custom intraoperative neurological monitoring (INM) software (Infinite Biomedical Technologies, Baltimore, MD) was used to set the stimulation parameters and trigger the stimulator. Positive voltage pulses of 3 V magnitude, 25 ms duration and 1 Hz frequency were used for limb stimulation. Somatosensory evoked potentials from the transcranial electrodes were amplified by an optically isolated biopotential amplifier (Opti-Amp 8002, Intelligent Hearing Systems, Miami, FL) with a gain of 30,000. The analog signal from each hemisphere was transferred to a personal computer via an optical data acquisition system with four input channels at a sampling rate of 5000 Hz. The SSEP signals, the stimulation pulse signal and the stimulated limb were recorded on separate channels for (post-operative) data analysis. The signal-to-noise ratio (SNR) was improved by ensemble averaging of 100 sweeps, with the averaging window shifting by 20 sweeps each time. To account for baseline differences across animals, each animal’s recordings were standardized to the baseline. Mean differences in each of these outcomes were examined with a repeated-measures analysis of variance (ANOVA). All statistical analyses were conducted with SPSS18.0 software (IBM Corporation, NY, USA) and P-values < 0.05 were considered statistically significant.

### Sensory function test

Thermal stimuli were assessed using the tail withdrawal latency to radiant heat according to the protocol of the tail-flick test. Animals were restrained in a plexi glass tube and placed on the tail-flick apparatus. A light beam was focused on the last third of the tail. The latency to remove the tail from the heat was recorded. The time when positive responses appeared was considered the tail withdrawal latency (TWL). Parameter setting: intensity 23 W, 16.0 s cut-off with 5 min intervals between trials. Tests were performed 4 weeks after miR-127 agomir delivery.

### Tissue harvest for histomorphological analysis

Spinal cord tissues that were collected 3 days post-operation (dpo) were used for TUNEL staining. And tissues collected 28 dpo were used for immunochemistry and *in situ* hybridization. In parallel experiments, animals were deeply anaesthetized with 3.6% chloral hydrate (100 mg/kg, i.p.) and transcardially perfused with heparinized physiological saline followed by 4% paraformaldehyde in 0.1 M ice-cold phosphate buffer, pH 7.4. Spinal cords were dissected at approximately 10 mm rostral to the injured epicenter. The spinal cord samples were post-fixed for 5 h at 4 °C. The tissue was successively soaked in 10%, 20% and 30% sucrose in 0.1 M phosphate buffer, pH 7.4, for 72 h at 4 °C. Then, the rostral segments of transected spinal cords from different groups were embedded in the same paraffin. The paraffin-embedded sections were cut into 5 μm slices and processed simultaneously. For immunocytochemical analyses of neurons, spinal neurons were cultured on glass coverslips in 6-well plates. Following washes in PBS, the cells were rinsed and fixed with 4% paraformaldehyde at room temperature for 30 min.

### *In situ* hybridization to test the localization of miR-127 in spinal cord

Sections were de-waxed in xylene, rehydrated in graded alcohols, and placed in diethyl pyrocarbonate (DEPC) H_2_O. Endogenous peroxidase was inactivated by incubation in 3% H_2_O_2_ for 15 min at RT. Sections were then digested in proteinase K (20 μg/ml, Sigma) for 20 min, rinsed in NaCl/Tris, and fixed in 4% PFA for 10 min. Following this, the slides were rinsed twice with PBS for 5 min. Slices were blocked at RT for 2 h in hybridization buffer (50% formamide, 25% 5 × saline sodium citrate (SSC), 10% 5 × Denhardt’s and 15% DEPC-H_2_O containing 200 ng/mL yeast RNA, 500 g/mL salmon sperm DNA and 20 mg/mL Roche blocking reagent) and then hybridized with 30 nmol of a locked nucleic acid (LNA)-modified oligonucleotide probe (Exiqon, Woburn, MA) complementary to *Rattus norvegicus* (rno) miR-127 and labeled with digoxigenin (D-D *In situ* hybridization detection kits, FOCO, Lot No.D-2204B) at 52 °C overnight. After hybridization, the slides were washed twice in 2 × SSC at 37 °C for 15 min, followed by one wash in 0.5 × SSC (15 min at 37 °C) and one wash in 0.2 × SSC (15 min at 37 °C). The slides were then incubated with HRP-conjugated anti-DIG antibody. The sections were rinsed three times in PBS for 5 min, and peroxidase staining was visualized with DAB for 3 min.

### Immunofluorescence staining of neural regeneration indicators *in vivo*

Spinal cords were subjected to immunohistochemical analysis of NeuN (a marker of neurons), GFAP (a marker of astrocytes), growth associated protein 43 (GAP-43, a marker for neural regeneration)^45^,^46^ and calcitonin gene related protein (CGRP, a marker of primary sensory neuron plasticity)^47^. And these markers were described previously for anterior and dorsal horn staining^48^,^49^,^50^,^51^,^52^,^53^. The spinal cords were cut into coronal sections and mounted on a gelatin-coated slide (Sigma). After being routinely de-paraffinized and rehydrated, the slices were permeated in PBS containing 3% goat serum (Golden Bridge, Beijing) for 30 min at 37 °C. Then, sections were incubated overnight at 4 °C with the primary antibody (see Table 2). The next day, the sections were washed three times with PBS and incubated with species-specific secondary antibodies for 30 min at 37 °C (see Table 2). The sections were washed three times with PBS and then cover slipped with Vecta shield mounting medium containing DAPI (4′,6-diamidino-2-phenyl-indole) to counterstain the nuclei. Species-specific non-immune IgG was used as a negative control for each antibody. Then, the images were obtained using a fluorescent microscope (Leica, Germany).

The percentage of NeuN/DAPI and the mean density of GFAP, GAP-43 and CGRP were quantified in every subject in a series of one-in-twenty immunolabeled 5-μm-thick coronal sections. Quantification was performed separately within grafts and in host spinal cord blocks located rostral to the lesion site at the following distances: 0–250, 250–500, 500–750, 750–1000 and 1000–1250 μm. For each slice, low-magnification photomicrographs (200×) were obtained to manually calculate the NeuN^+^ cells, which were averaged with five anterior and dorsal horn sections per animal and presented as the percentage of NeuN/DAPI. Additionally, high- magnification (400×) images were employed to measure the mean density of GFAP, GAP-43 and CGRP. Images of the white matter were obtained to measure the density of GFAP. Images of the anterior, intermediate zone and dorsal horn per lamina in the spinal cord were obtained to measure the density of GAP-43. The medial, middle and lateral regions of the lamina I/II were scanned to calculate the density of CGRP. The mean density was presented as IOD (integrated optical density) over the total (mm^2^) area using Image-Pro Plus 6.0 software (MediaCybernetics, Silver Spring, MD, USA) as described previously^54^. All the detection was evaluated by three investigators blinded to the experimental information.

### Bioinformatics prediction

To investigate how miR-127 participates in the regulation of neural regeneration, we sought to identify miR-127 target genes in primary spinal neurons. Target genes of miR-127-3p were predicted with four algorithms: TargetScan v.6.2, miRNAWalk, miRDB and miRGen with a highly stringent score cutoff of ≥140 and a minimum free energy cutoff of ≤−18.0. The genes in the predicted target list were then filtered based on their overlap within each database. Three common targets, MitoNEET, KCC1 and Spock2, were chosen for further study.

### Luciferase Activity Assays

MitoNEET, KCC1 and Spock2 3′ UTR luciferase plasmids as well as the corresponding mutant plasmids were generated by RiboBio (GuangZhou, China). The pmiR-RB-REPORT™ Dual luciferase-expressing vector contained hRlucc DNA encoding *Renilla* luciferase as a reporter and hLucc DNA encoding firefly luciferase as an internal control. Constructs of luciferase plasmid contained the full-length 3′-UTRs of MitoNEET, KCC1 and Spock2 mRNA, and the mutant plasmids of MitoNEET and KCC1 contained a 3′ UTR mutation known to effectively abrogate the binding of MitoNEET and KCC1 to miR-127. Because there was no binding site between the 3′ UTR of Spock2 mRNA and miR-127, the 3′ UTR mutation of Spock2 was not constructed. To create the reduced miR-127 affinity, the “GGAUCCG” bases of mitoNETT and KCC1 were mutated to “CCTAGGC”. The constructs were confirmed by XhoI and NotI (Promega) restriction enzyme digestion and sequencing. Then, 293Tα cells (5 × 10^5^ cells per well) were seeded into triplicate wells of 6-well plates and allowed to settle for 12 h. The 3′ UTR luciferase plasmids of MitoNEET, KCC1 and Spock2 or control reporter plasmid (100 ng/ml, Guangzhou RiboBio, China) were transfected into 293Tα cells using SuperFectinTM II *In Vitro* DNA Transfection Reagent (Pufei Biotech, China). Cells were harvested 48 h after transfection, and luciferase activity was measured with a Dual-Luciferase Reporter Assay Kit (Promega, E1910).

### Detection for the localization of MitoNEET and KCC1

Double immunofluorescence staining of NeuN and MitoNEET or KCC1 and of GFAP and MitoNEET or KCC1 was performed to detect the origins of MitoNEET and KCC1. Briefly, the dorsal horn of spinal cord tissues were cut into coronal sections and being routinely de-paraffinized and rehydrated, then, the slices were permeated in PBS containing 3% goat serum for 30 min at 37 °C as previously described. Subsequently, the sections were incubated with the appropriate primary antibodies and species-specific secondary antibodies (see Table 3).

### Culture and identification of primary spinal neurons

Spinal neurons were obtained from one-day-old neonatal SD rats following a previously reported protocol^55^. Briefly, neonatal SD rats were decapitated at the base of the foramen magnum after sterilization. The spinal cords were harvested and cut into approximately 1 mm^3^ small pieces, then digested with 0.05% trypsin (Gibco) at 37 °C for 10 min and eluted with 10% BSA (bovine serum albumin, Gibco). The tissue suspension was centrifuged at 1000 rpm for 10 min. The pellets in the bottom were resuspended in complete culture medium (Hyclone) composed of DMEM/HIGH GLUCOSE, 10% fetal calf serum (Gibco) and 1% penicillin-streptomycin solution (Hyclone). Neurons were plated in 6-well plates (Corning, USA) coated with poly-d-lysine and laminin (Sigma-Aldrich, St. Louis, MO) at a density of 5 × 10^5^ cells/ml. Four hours after incubation at 37 °C and 5% CO_2_, the complete culture medium was replaced with neurobasal medium with the addition of 2% B27 (Invitrogen, Carlsbad, CA). One-half of the culture medium was changed every 3 days. The neuron population in the culture was quantified by counting cells with NeuN-positive staining after 3 days of culture as described previously.

### Real-Time Quantified PCR (qRT-PCR)

Total RNA was isolated with Trizol reagent (Takara Bio Inc., Otsu, Japan) and was reverse transcribed. The premier sequences for qRT-PCR were as follows: MitoNEET (forward) 5′-CCAGAAAGACAACCCGAAG-3′ and (reverse) 5′-GTGCTTTATGTG AGCCCCAT-3′; KCC1 (forward) 5′-CCATGTTCTTTCTGATGTGTT-3′ and (reverse) 5′-ACCAGGAGGAGACAAACAT-3′; β-actin (forward) 5′-GAAGATCAA GATCATTGCTCCT-3′ and (reverse) 5′-TACTCCTGCTTGCTGATCCA-3′. The rat β-actin housekeeping gene was used as an internal control. The PCR products were verified by 1% agarose gel electrophoresis and visualized with Goldview staining (WOLSEN). Finally, the gels were analyzed by Alpha Innotech (BIO-RAD), and the optical density (OD) was analyzed on a computer using ImageJ software.

### ELISA

Enzyme-linked Immunosorbent Assay Kit for MitoNEET (Cloud-clone Corp, SEL121Ra) and KCC1 (Cloud-clone Corp, SEE376Ra) were employed to detect the proteins in primary spinal neurons. At 3 days after being transfected with miR-127, primary spinal neurons were detached with trypsin and then collected by centrifugation. Dilutions (100 μL) of the standards and samples (at a total concentration of 1 μg/ml) were added to a 96-well plate and incubated for 2 h at 37 °C, followed by aspiration, the addition of 100 μL of prepared Detection Reagent A, incubation for 1 h at 37 °C, the addition of 100 μL of prepared Detection Reagent B, incubation for another 30 minutes at 37 °C, the addition of 90 μL of Substrate Solution and incubation for 15–25 minutes at 37 °C with 50 μL of Stop Solution/well. The protein concentration at 450 nm was read immediately.

### Transfection of miR-127 mimic/anti-miR-127 or siRNAs into primary spinal neurons

P1 spinal neurons were cultured for 7 days with cells at 50–80% confluence prior to transfection. For the transplantation of miRNAs, primary cultured spinal neurons were divided into four groups: normal (neurobasal medium only), NS-miRNA (nonsense miRNA), miR-127 mimic and anti-miR-127 group. For the transplantation of siRNAs, primary cultured spinal neurons were divided into four groups: normal (neurobasal medium only), NS-siRNA (nonsense siRNA), si-MitoNEET and si-KCC1 groups. MiR-127 mimic (Lot 1101), anti-miR-127 (Lot 0829) and siRNAs were designed and synthesized by RiboBio (Guangzhou, China). Three siRNAs targeting the MitoNEET and KCC1 genes were designed and synthesized, and the most effective siRNAs identified by qRT-PCR, si-MitoNEET (Lot 1022) and si-KCC1 (Lot 1022), were applied for further experiments. The sequence of si-MitoNEET was as follows: sense: (5′-3′) 5′UCGGUUACCUGGCUUACAAdTdT3′; antisense (3′-5′) 3′dTdTAGCCAAU GGACCGAAUGUU5′. The sequence of si-KCC1 was as follows: sense (5′-3′) 5′GCCUUGAACGGGUGUUGUUdTdT3′; antisense (3′-5′) 3′dTdTCGGAACUUGC CCACAACAA5′. Transfection was performed using SuperFectin^TM^ II *in vitro* siRNA transfection reagent (Pufei Biotech, China). Briefly, a mix of transfection stock Buffer and miRNA or siRNA was prepared and 3 μl of SuperFectin™ II reagent was added to the mixture. Mixture of miR-127 mimic (80 nM), anti-miR-127 (100 nM) or siRNA (100 nM) was added drop-wise to the appropriate wells, respectively. After incubation at 37 °C for 24 h, another 1.2 ml of fresh culture medium was added and not replaced. qRT-PCR was performed, and red Cy3-5′-fluorescence (RiBio, Lot N1230) was observed with a fluorescence microscope (Leica CM 1860, Germany) 72 h after transfection to confirm the knockdown of MitoNEET and KCC1.

### Immunofluorescence staining of neural regeneration indicators *in vitro*

Immunocytochemical analysis of NeuN and GFAP were performed to detect the effect of miR-127 transfection on the number of neurons and astrocytes. NeuN and GAP-43 immunocytochemical analysis were performed to detect the neural regeneration after si-MitoNEET administration. Briefly, for neuronal immunocytochemistry, slices were directly permeated in PBS containing 3% goat serum for 30 min at 37 °C. Then, the slices were incubated with the appropriate primary antibodies and species-specific secondary antibodies (see Table 4) as described previously. The number of NeuN^+^ cells and GFAP^+^ cells quantified *in vitro* was normalized to the total DAPI to confirm the purity of the neurons and astrocytes. Then number of NeuN^+^ cells and GFAP^+^ cells were counted per mm^2^ to compare the population changes of neurons and astrocytes in different groups. The mean density of GAP-43 was calculated as previously described. Data are presented as the means ± SEM. All the detection was evaluated by 3 investigators blinded to the experimental information.

### Measurement of axon length in neurons

Three and five days after the transfection of miR-127 mimic/anti-miR-127 or siRNAs (si-MitoNEET and si-KCC1), images of these neurons were obtained under bright-field microscopy with a Leica AF6000 cell station (CM8600, Leica Microsystems, Buffalo Grove, IL, USA). For measurements of the axon length of living neurons only, images with a magnification of 200× were randomly selected. Axon length was measured with LAS AF software (Leica Microsystems) and the lengths of 150–200 single neurons per condition were quantified using LAS AF software. The mean axonal length was calculated from counts on a 6-well plate in each group with 5 images in each well, which were obtained from the upper, middle, lower, left and right of each well respectively. And five wells in each group were used. Three examiners who can distinguish the axon and dendrite easily were employed to measure the axon length, and all the examiners were blinded to the group identity.

### TUNEL assay to test apoptotic cells *in vitro* and *in vivo*

A TUNEL reaction mixture of enzyme solution and labeling solution (*In Situ* Cell Death Detection Kit, TMR red; Cat. NO. 12156792910) was added at a ratio of 1:9 (v/v), and the slices were stored at 4 °C overnight in the dark. After three washes with PBS, the slices were stained with DAPI for 5 min at room temperature, and photographs were obtained under fluorescence microscopy (Leica, CM1860, Germany). After five fields were randomly selected from each section, each field was measured by an observer who did not identify the slides. Apoptosis was quantified by determining the percentage of TUNEL/DAPI using Image-Pro Plus 6.0 software.

### Statistical Analysis

Samples for cell growth, qRT-PCR analysis and promoter luciferase assays were run in triplicate. The data were analyzed using one-wayANOVA or repeated-measure ANOVA. If equal varianceswere found, Fisher’s least significant difference test wasperformed. Otherwise, the Kruskal-Wallis Test and Dunnett’s T3 were used. All statistical analyses were performed with SPSS18.0 software (IBM Corporation, NY, USA). P < 0.05 was considered statistically significant.

## Additional Information

**How to cite this article**: He, Q.-Q. *et al.* MicroRNA-127 targeting of mitoNEET inhibits neurite outgrowth, induces cell apoptosis and contributes to physiological dysfunction after spinal cord transection. *Sci. Rep.* **6**, 35205; doi: 10.1038/srep35205 (2016).

## Supplementary Material

Supplementary Information

## Figures and Tables

**Table 1:** Experiments Design (Different Parameters Measured).

Experiment 1		Experiment 2					
Spinal cord		Spinal cord				Spinal cord neurons	
Model	3 dpo	Group	3 dpo	28 dpo	Behavior test	Group	3 dpo
Sham SCT	MiRNA array (n = 3/group)	Sham SCT NS-miRNA miR-127	TUNEL (n = 5/group)	IHC; ISH (n = 5/group)	BBB score for 9 weeks; Tail-flick; SSEPs (n = 5 at least/group)	Normal NS-miRNA miR-127 anti-miR127 or Normal NS-siRNA si-mitoNEET si-KCC1	TUNEL/ICC (n = 5/group)
	Confirmation of microarray data– qRT-PCR (0 dpo, 3 dpo, 5 dpo n = 5/group)						

Sham: animals were only subjected to laminectomy; SCT: spinal cord transection; NS-miRNA: spinal cord transection with microinjection of miR-127 agomir negative control; miR-127: spinal cord transection with microinjection of miR-127 agomir; TUNEL: Terminal Deoxynucleotidyl Transferased Utp Nick End Labeling (TUNEL) staining; IHC: Immunocytochemistry staining; ISH: *In situ* hybridization for determining the location of miR-127 in spinal cord; BBB score: Basso, Beattie, and Bresnehan; Tail-flick: tail withdrawal latency to radiant heat; SSEPs: Somatosensory Evoked Potentials recording; ICC: Immunocytochemistry; NS-miRNA: negative control of miR-127 mimic/inhibitor; NS-siRNA: negative control of siRNA; dpo: days post-operation.

**Table 2:** Information of the primary antibodies and the respective secondary antibodies.

Primary antibody	Species	Source	Dilution	Lot.	Secondary antibodies	Dilution	Source
GAP-43	Mouse	SANTA CRUZ	1:50	Sc-33705	Cy3 anti-mouse	1:200	Abcam
NeuN	Mouse	Golden Bridge	1:100	ZM-0352	Cy3 anti-mouse	1:200	Abcam
CGRP	Rabbit	Bioss	1:100	Bs-0791R	Cy3 anti-Rabbit	1:200	Abcam
GFAP	Rabbit	Golden Bridge	1:50	ZA-0117	Cy3 anti-Rabbit	1:200	Abcam

**Table 3:** Information of the primary antibodies and the respective secondary antibodies.

Primary antibody	Species	Source	Dilution	Lot.	Secondary antibodies	Dilution	Source
MitoNEET	Mouse	Abcam	1:100	ab190166	Cy3 anti-mouse	1:200	Abcam
NeuN	Rabbit	Abcam	1:100	ab177487	Alexa fluor 488 anti-Rabbit	1:200	Abcam
GFAP	Rabbit	Golden Bridge	1:50	ZA-0117	Alexa fluor 488 anti-Rabbit	1:200	Abcam
KCC1	Rabbit	Abcam	1:100	ab188651	Cy3 anti-rabbit	1:200	Abcam
NeuN	Mouse	Golden Bridge	1:100	ZM-0352	Alexa fluor 488 anti-Mouse	1:200	Abcam
GFAP	Mouse	Golden Bridge	1:100	TA500335	Alexa fluor 488 anti-Mouse	1:200	Abcam

**Table 4:** Information of the primary antibodies and the respective secondary antibodies.

Primary antibody	Species	Source	Dilution	Lot.	Secondary antibodies	Dilution	Source
GAP-43	Mouse	SANTA CRUZ	1:50	Sc-33705	Cy3 anti-mouse	1:200	Abcam
NeuN*	Mouse	Golden Bridge	1:100	ZM-0352	Cy3 anti-mouse	1:200	Abcam
NeuN#	Mouse	Golden Bridge	1:100	ZM-0352	Alexa fluor-488 anti-mouse	1:200	Abcam
GFAP	Rabbit	Golden Bridge	1:50	ZA-0117	Cy3 anti-Rabbit	1:200	Abcam

^*^Represents the immunocytochemical test after miR-127 administration *in vitro*.

^#^Represents the immunocytochemical test after si-MitoNEET and si-KCC1 administration *in vitro*.
