# Erratum: MicroRNA-127 targeting of mitoNEET inhibits neurite outgrowth, induces cell apoptosis and contributes to physiological dysfunction after spinal cord transection

**DOI:** 10.1038/srep39570

**Published:** 2017-01-19

**Authors:** Qin-Qin He, Liu-Lin Xiong, Fei Liu, Xiang He, Guo-Ying Feng, Fei-Fei Shang, Qing-Jie Xia, You-Cui Wang, De-Lu Qiu, Chao-Zhi Luo, Jia Liu, Ting-Hua Wang

Scientific Reports 6: Article number: 35205; 10.1038/srep35205 published online: 10 17 2016; updated: 01 19 2017.

The original version of this Article contained a typographical error in Affiliation 1. The affiliation:

Institute of Neurological Disease, Department of Anesthesiology and Translational Neuroscience Center, the state key laboratory of Biotherapy, West China Hospital, Sichuan University, Kunming 650500, China.

now reads

Institute of Neurological Disease, Department of Anesthesiology and Translational Neuroscience Center, the state key laboratory of Biotherapy, West China Hospital, Sichuan University, Chengdu 610041, China.

In addition the Acknowledgements section in this Article is incomplete.

“We thank Dr. Zhi-cheng Xiao for discussion and critical comments, Dr. Visar for close editing of the manuscript.”

now reads

“We thank Dr. Zhi-cheng Xiao for discussion and critical comments, Dr. Visar for close editing of the manuscript. This research was supported by a Grant of National Science Foundation of China, No. 81271358, and a Grant of National Science Foundation of China, No. 81271358, and a Grant of National Science Foundation of China, No. 81471268. This study was also supported by the Program for IRTSTYN, together with the program Innovative Research Team in Science and Technology in Yunnan province”.

These errors have been corrected in the HTML and PDF versions of this Article.

