# Supplementary material for: MicroRNA-127 targeting of mitoNEET inhibits neurite outgrowth, induces cell apoptosis and contributes to physiological dysfunction after spinal cord transection
Source: Sci Rep. 2016 Oct 17;6:35205. doi: 10.1038/srep35205 (PMC5066253; doi:10.1038/srep35205)
Supplement: Supplementary Information [file srep35205-s1.doc]

**MicroRNA-127 targeting of mitoNEET inhibits neurite outgrowth, induces cell apoptosis and contributes to physiological dysfunction after spinal cord injury**

Qin-Qin He^1*^, Liu-Lin Xiong^1*^, Fei-Liu^1^, Xiang-He^1^, Guo-Ying Feng^3^, Fei-Fei Shang^4^, Qing-Jie Xia^1^, You-Cui Wang^4^, De-Lu Qiu^1^, Chao-Zhi Luo^1^, Jia-Liu ^2$^, Ting-Hua Wang^1, 2, 3$^

^1^Institute of Neurological Disease, Department of Anesthesiology and Translational Neuroscience Center, West China Hospital, Sichuan University, 610041 Chengdu, China

^2^Institute of Neuroscience, Animal Zoology Department, Kunming medical University, Kunming, Yunnan, 610041, China

^3^Department of Histology and Embryology, West China School of Preclinical and Forensic Medicine, State key lab of biotherapy, Sichuan University, Chengdu 610041, China.

^4^ Institute of Neurological Disease, the state key laboratory of Biotherapy, Department of Collaborative Innovation Center for Biotherapy, West China Hospital, Sichuan University, Chengdu, 61041, P.R. China.

*, ^$^ These authors contributed equally to this work.

Corresponding Author:

Jia-Liu

Institutes of Neuroscience, Animal Zoology Department, Kunming medical University, Kunming, Yunnan, 610041, China

Email: [liujiaaixuexi@126.com](mailto:liujiaaixuexi@126.com)

Ting-Hua Wang

Department of Anesthesiology, Institute of Neurological Disease, Translational Neuroscience Center, West China Hospital, Sichuan University, 610041 Chengdu, China.

Email: [tinghua_neuron@263.net](mailto:tinghua_neuron@263.net)

Tel/Fax: 86-28-85501036

|  |
| --- |
|
|


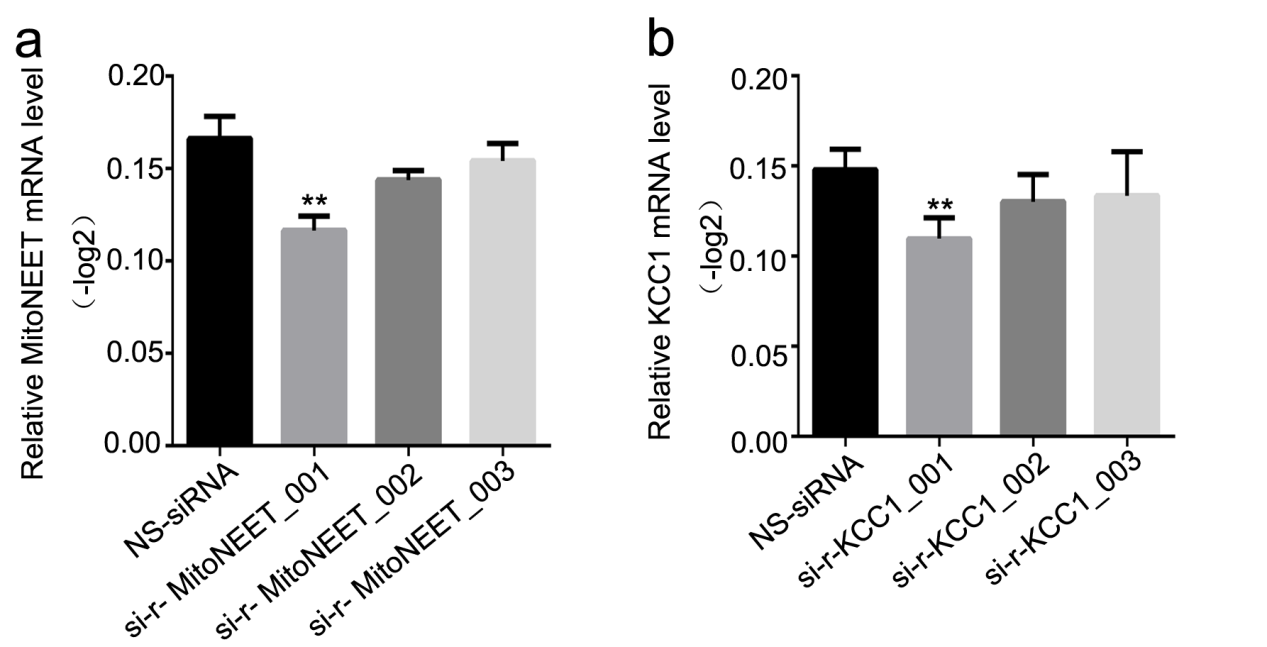


**Supplementary Fig.S1** | Screening of the most effective small interference RNAs of MitoNEET and KCC1. (a)Total RNA of PC12 cells was extracted and qRT-PCR of MitoNEET was performed 72 hours after being transfected with three fragments of si-MitoNEET. (b) Total RNA of PC12 cells was extracted and qRT-PCR of KCC1 was performed 72 hours after being transfected with three fragments of si-KCC1. *P<0.05, **P<0.01, compared with NS-siRNA.


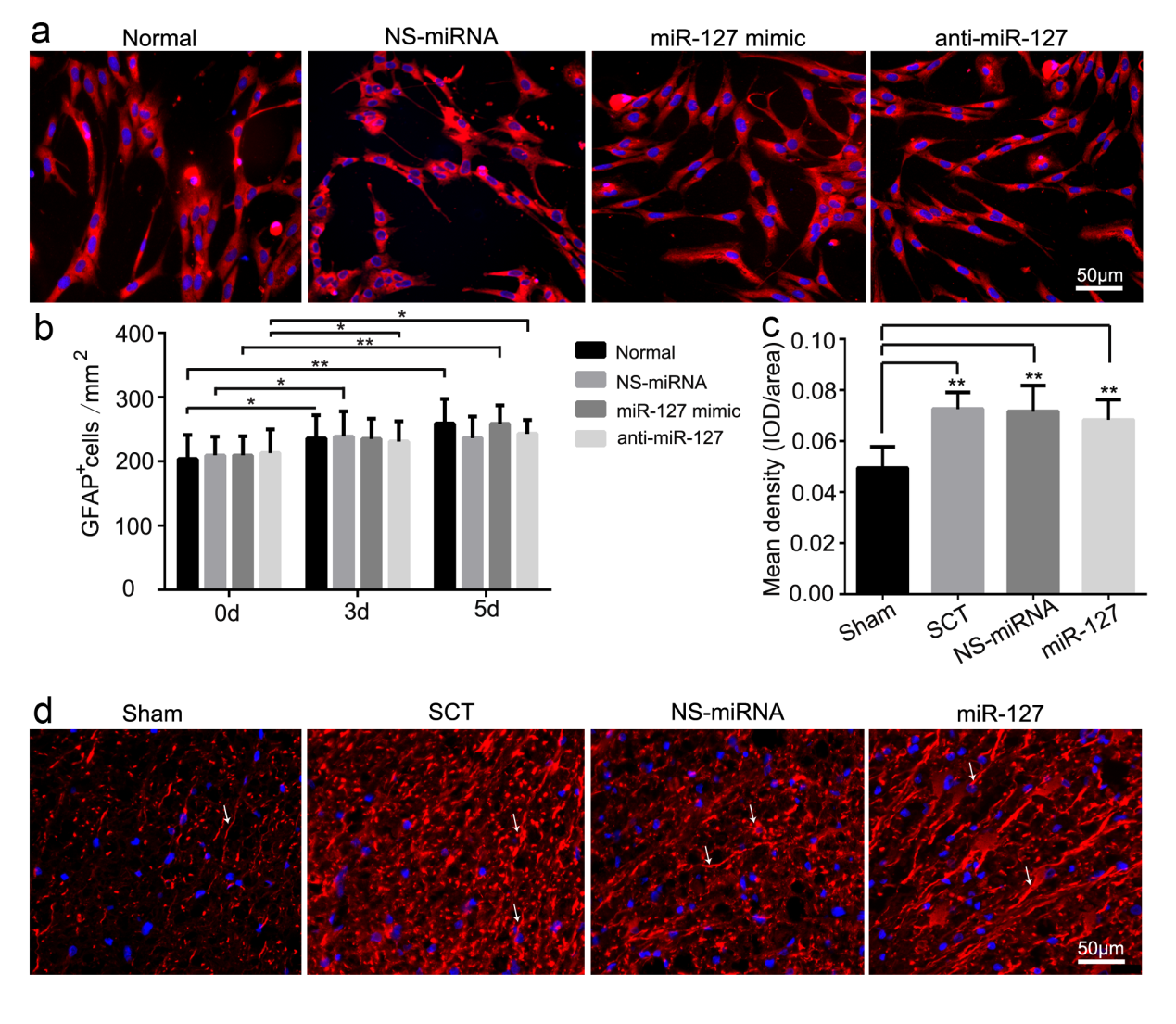


**Supplementary Fig.S2** | MiR-127 displayed no effect on the population of astrocytes. (a) GFAP immunoreactive staining in normal, NS-miRNA (80nM), miRNA-127 mimic (80nM) and anti-miR-127 (100nM) group. Red signal represented GFAP- positive cells and blue signal represented nucleus of all cell types. (b) Average number of GFAP positive cells per mm^2^ after being transfected with miR-127 mimic or anti-miR-127 was measured. Graphs represent the mean ± SEM of quintuplicate culture dishes per group at each time point from three separate experiments. *P<0.05, **P<0.01, compared at different time point in the same group. #P<0.05, ## P<0.01 compared with the NS-miRNA group at the same time point. (c) Mean density of GFAP, which presented as IOD/Area in each group were measured in sham group, SCT, NS-miRNA and miR-127 group. *P<0.05, **P<0.01. (d) Slices of rostral spinal cord derived from 28 days after operation were subjected to immunohistochemistry for GFAP (white arrow) in sham, SCT, NS-miRNA and miR-127 group. Scale bar: a,d,50 μm.


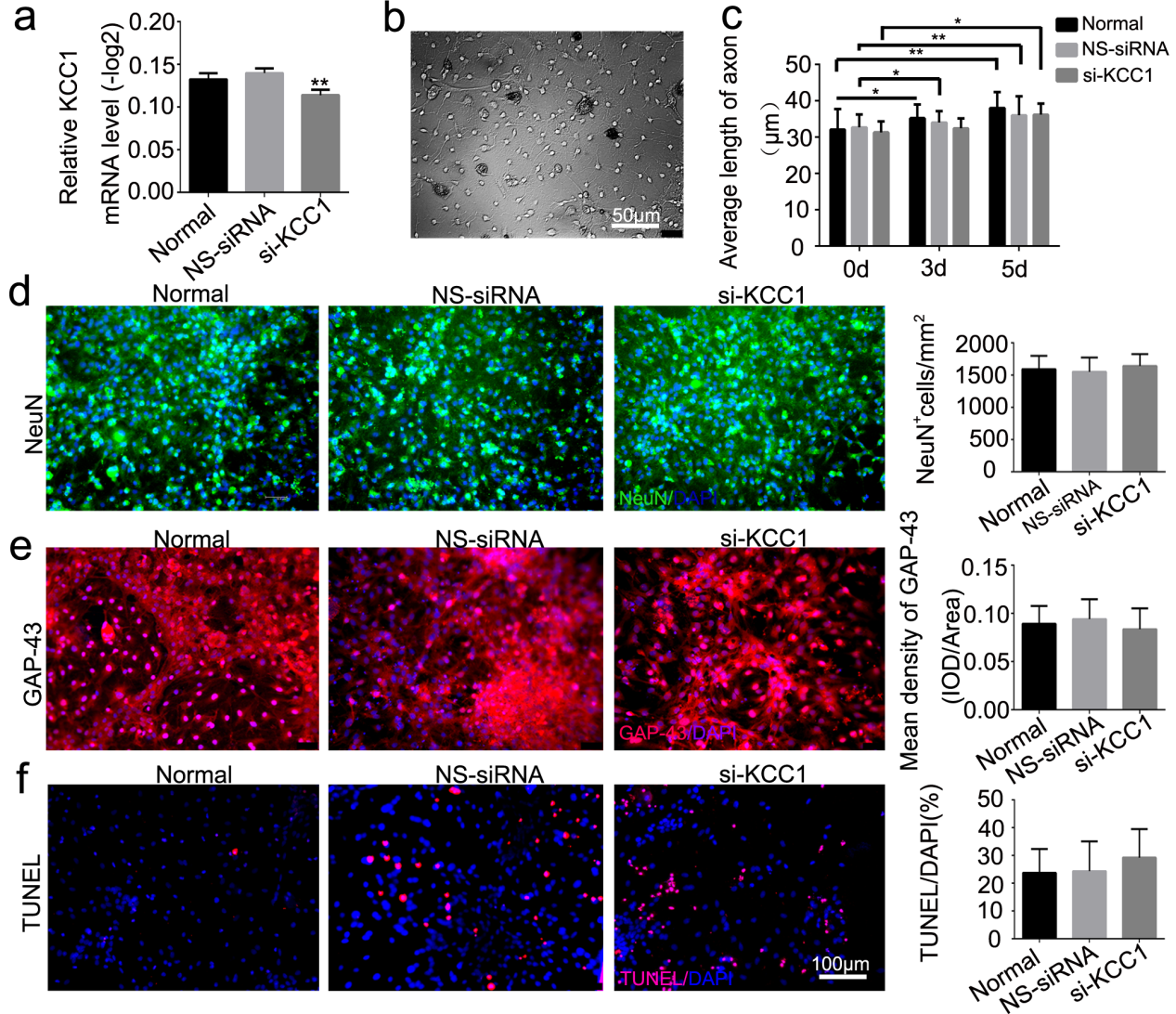


**Supplementary Fig.S3** | Knock down of KCC1 existed no effected on the population, axonal length, and neural apoptosis in primary cultured spinal cord neurons. (a) Total RNA of primary cultured spinal cord neurons was extracted and qRT-PCR of KCC1 was performed 72 hours after being transfected with si-KCC1 and NS-siRNA. (b) Representative bright field picture of the primary cultured spinal neurons at 5 days after being transfected with NS-siRNA. (c)Average length of axon in Normal, NS-siRNA, and si-KCC1 group was measured by using Leica AF6000 cell station. Graphs represent the mean ± SEM of quintuplicate culture dishes per group at each time point from three separate experiments. *P<0.05, **P<0.01, compared at different time point in the same group. (d) Immunostaining of NeuN in normal, NS-siRNA (100nM) and si-KCC1 (100nM) group. Green signal represented NeuN-positive neuron and blue signal represented nucleus of all cell types (left). And average number of NeuN positive cells per mm^2^ was measured (upper right). (e) Immunofluorescence staining of GAP-43 (red,left) in normal, NS-siRNA and si-KCC1 group was performed. DAPI (blue) was employed to show all nuclei. Mean density of GAP-43, which presented as IOD/Area in each group was measured (upper right). (f) TUNEL staining (white arrow) was performed in normal, NS-siRNA and si-KCC1 group .DAPI (blue) was used to show all nuclei. The percent of TUNEL/DAPI was evaluated and indicated by quantitative histogram (upper right). *P<0.05, **P<0.01, compared with NS-siRNA. Scale bar: b,50 μm;d-f,100 μm.

**Supplementary table**

| **Comparision of the miRNAs in our experiment with other previously reported miRNAs** | | | | | | | | | | |
| --- | --- | --- | --- | --- | --- | --- | --- | --- | --- | --- |
|  |  |  |  |  |  |  |  |  |  |  |
| Author | Present study | | | Mónica Yunta | | | Jian-Zhong Hu | | Nakanishi K | |
|
| Model | Completed SCT (T10) | | | Moderate contusion (200 kdyne)(T8) | | | Contusion,Allen Weight Drop (8 g, 40 mm) T10 | | Compressing the cord laterally from both sides for 10 s (T11–12) | |
|
|
|
| Animal | rat | | | rat | | | rat | | mouse | |
| Group | S | SCT | P/A | CT | IN3 | SH3 | SH3 | IN3 | CT | IN3 |
| miRNA | The comparision data | | | | | | | | | |
| let-7d* | X | X↓ | P |  | X | X |  |  |  |  |
| miR-10a | X | X↓ | A |  |  |  |  |  |  |  |
| miR-124 | X | X↓ | P | X | X | X |  |  | X | X↓ |
| miR-124* | X | X↓ | A |  |  |  |  |  |  |  |
| miR-127 | X | X↓ | A |  |  |  |  |  |  |  |
| miR-134 | X | X↓ | A |  |  |  |  |  |  |  |
| miR-136* | X | X↓ | A |  |  |  |  |  |  |  |
| miR-199a-5p | X | X↓ | A |  |  |  |  |  |  |  |
| miR-192 | X | X↓ | A |  |  |  |  |  |  |  |
| miR-215 | X | X↓ | A |  |  |  |  |  |  |  |
| miR-30b-5p | X | X↓ | P | X | X | X |  |  |  |  |
| miR-30c-1* | X | X↓ | P | miR-30c | X | X |  |  |  |  |
| miR-101a* | X | X↓ | A |  |  |  |  |  |  |  |
| miR-208 | X | X↓ | A |  |  |  |  |  |  |  |
| miR-291a-3p | X | X↓ | A |  |  |  |  |  |  |  |
| miR-376a | X | X↓ | A |  |  |  |  |  |  |  |
| miR-376b-5p | X | X↓ | A |  |  |  |  |  |  |  |
| miR-324-3p | X | X↓ | A |  |  |  |  |  |  |  |
| miR-326 | X | X↓ | A |  |  |  |  |  |  |  |
| miR-330* | X | X↓ | A |  |  |  |  |  |  |  |
| miR-345-3p | X | X↓ | A |  |  |  |  |  |  |  |
| miR-347 | X | X↓ | A |  |  |  |  |  |  |  |
| miR-350 | X | X↓ | A |  |  |  |  |  |  |  |
| miR-369-3p | X | X↓ | A |  |  |  |  |  |  |  |
| miR-380 | X | X↓ | A |  |  |  |  |  |  |  |
| miR-410 | X | X↓ | P |  |  |  | X | X↓ |  |  |
| miR-421 | X | X↓ | A |  |  |  |  |  |  |  |
| miR-431 | X | X↓ | A |  |  |  |  |  |  |  |
| miR-471 | X | X↓ | A |  |  |  |  |  |  |  |
| miR-489 | X | X↓ | A |  |  |  |  |  |  |  |
| miR-532 | X | X↓ | A |  |  |  |  |  |  |  |
| miR-541 | X | X↓ | A |  |  |  |  |  |  |  |
| miR-598-5P | X | X↓ | A |  |  |  |  |  |  |  |
| miR-671 | X | X↓ | A |  |  |  |  |  |  |  |
| miR-760-5p | X | X↓ | A |  |  |  |  |  |  |  |
| miR-770 | X | X↓ | A |  |  |  |  |  |  |  |
| miR-880 | X | X↓ | A |  |  |  |  |  |  |  |
| miR-9* | X | X↓ | P | X | X | X |  |  |  |  |
| miR-92a | X | X↓ | A |  |  |  |  |  |  |  |
| miR-99b | X | X↓ | A |  |  |  |  |  |  |  |
| let-7a |  |  |  | X | X | X |  |  |  |  |
| let-7b |  |  |  | X | X | X | X | X↓ |  |  |
| let-7c |  |  |  | X | X | X |  |  |  |  |
| let-7e |  |  |  |  |  | X |  |  |  |  |
| let-7f |  |  |  | X | X | X |  |  |  |  |
| let-7g |  |  |  | X | X | X |  |  |  |  |
| let-7i |  |  |  |  | X | X |  |  |  |  |
| miR-10b |  |  |  |  |  |  |  |  |  |  |
| miR-125b-5p |  |  |  | X | X | X |  |  |  |  |
| miR-126 |  |  |  | X | X | X | X | X↓ |  |  |
| miR-1280 |  |  |  | X | X | X |  |  |  |  |
| miR-1308 |  |  |  | X | X | X |  |  |  |  |
| miR-135a |  |  |  |  |  |  |  |  |  |  |
| miR-142-3p |  |  |  |  | X |  |  |  |  |  |
| miR-143 |  |  |  | X |  | X |  |  |  |  |
| miR-144 |  |  |  |  | X |  |  |  |  |  |
| miR-16 |  |  |  | X | X | X |  |  |  |  |
| miR-17-5p |  |  |  |  |  |  | X | X↑ |  |  |
| miR-1826 |  |  |  | X | X | X |  |  |  |  |
| miR-1827 |  |  |  | X | X | X |  |  |  |  |
| miR-21 |  |  |  |  | X |  | X | X↑ |  |  |
| miR-219-5p |  |  |  | X | X | X |  |  |  |  |
| miR-22 |  |  |  | X | X | X |  |  |  |  |
| miR-223 |  |  |  |  |  |  |  |  | X | X↑ |
| miR-23a |  |  |  |  | X | X |  |  |  |  |
| miR-23b |  |  |  | X | X | X |  |  |  |  |
| miR-24 |  |  |  | X | X | X | X | X↓ |  |  |
| miR-26a |  |  |  | X | X | X |  |  |  |  |
| miR-26b |  |  |  | X | X | X |  |  |  |  |
| miR-27b |  |  |  |  |  |  | X | X↓ |  |  |
| miR-29a |  |  |  | X | X | X |  |  |  |  |
| miR-29b |  |  |  | X |  | X |  |  |  |  |
| miR-30a |  |  |  | X | X | X |  |  |  |  |
| miR-451 |  |  |  | X | X | X |  |  |  |  |
| miR-467e* |  |  |  | X |  |  |  |  |  |  |
| miR-690 |  |  |  | X | X | X |  |  |  |  |
| miR-709 |  |  |  | X | X | X |  |  |  |  |
| miR-720 |  |  |  | X | X | X |  |  |  |  |
| miR-9 |  |  |  | X |  | X |  |  |  |  |
| miR-923 |  |  |  | X |  |  |  |  |  |  |
| miR-93 |  |  |  |  |  |  |  |  |  |  |
| miR-96 |  |  |  |  |  |  |  |  |  |  |
| miR-98 |  |  |  |  |  |  |  |  |  |  |
| miR-99a |  |  |  |  |  |  | X | X↓ |  |  |
| miR-3591 |  |  |  |  |  |  | X | X↑ |  |  |
| miRPlus-E1013 |  |  |  | X | X | X |  |  |  |  |
| miRPlus-E1024 |  |  |  | X | X | X |  |  |  |  |
| miRPlus-E1038 |  |  |  |  |  |  |  |  |  |  |
| miRPlus-E1078 |  |  |  | X | X | X |  |  |  |  |
| miRPlus-E1100 |  |  |  | X | X | X |  |  |  |  |
| miRPlus-E1103 |  |  |  | X | X | X |  |  |  |  |
| miRPlus-E1117 |  |  |  | X | X |  |  |  |  |  |
| miRPlus-E1218 |  |  |  | X | X | X |  |  |  |  |
| miRPlus-E1252 |  |  |  | X | X | X |  |  |  |  |
| miRPlus-E1253 |  |  |  | X | X | X |  |  |  |  |
| miRPlus-E1258 |  |  |  | X | X |  |  |  |  |  |
| miRPlus-E1290 |  |  |  | X | X | X |  |  |  |  |
| miRPlus-F1003 |  |  |  | X | X | X |  |  |  |  |
|  |  |  |  |  |  |  |  |  |  |  |
| Data from our analysis are shown in present study marked with X. | | | | | | | | |  |  |
| Other right three colums indicate thedifferent microRNAs in the previous microarray analysis. | | | | | | | | | | |
| P represents the microRNA detected both in our experiment and in the previous study | | | | | | | | | | |
| A represents the microRNA detected in our experiment but not in the previous study | | | | | | | | | |  |
| X indiaced the related name for the reported miRNA. | | | | | | | |  |  |  |
| ↑/↓indicated miRNA was up-regulated or down-regulated after spinal cord injury. | | | | | | | | | |  |
| Yellow represents the miRNAs in our microRNA array analysis | | | | | | | |  |  |  |
| Green reprensents the miRNAs both in our microRNA array analysis and other previous studies | | | | | | | | | | |
| CT: Control | SH3: 3 days after the sham operation | | | | | | |  |  |  |
| IN3: 3 days after injury | | |  |  |  |  |  |  |  |  |
| SCT:transected spinal cord injury | | | | |  |  |  |  |  |  |
| S：Sham |  |  |  |  |  |  |  |  |  |  |
|  |  |  |  |  |  |  |  |  |  |  |
|  |  |  |  |  |  |  |  |  |  |  |
